# Supplementary material for: Enhancing Efficiency of Natural Product Structure Revision: Leveraging CASE and DFT over Total Synthesis
Source: Molecules. 2023 Apr 28;28(9):3796. doi: 10.3390/molecules28093796 (PMC10180399; doi:10.3390/molecules28093796)
Supplement: Supplementary file 1 [file molecules-28-03796-s001.zip › molecules-2358296-supplementary.pdf]

Supplementary Materials

Enhancing Efficiency of Natural Product Structure Revision: Leveraging CASE and DFT over Total Synthesis

Mikhail Elyashberg [1,*], Sriram Tyagarajan [2], Mihir Mandal [2], and Alexei V. Buevich [3,*]

[1] Advanced Chemistry Development Inc. (ACD/Labs), Toronto, ON, M5C 1B5 Canada; mikhail.elyashberg@acdlabs.com
[2] Medicinal Chemistry, Merck & Co., Inc., Kenilworth, New Jersey 07033, United States; alexei.buevich@merck.com
[3] Analytical Research and Development, Merck & Co., Inc., Kenilworth, New Jersey 07033, United States; alexei.buevich@merck.com
*Correspondence: mikhail.elyashberg@acdlabs.com, alexei.buevich@merck.com

Contents

Table S1. NMR spectroscopic data of Macahydantoin B [1] 3

Table S2. NMR spectroscopic data of clionastatin B [2] 4

Figure S1. MCD for clionastatin B. 5

Table S3. NMR spectroscopic data of pyrostatin B [3] 6

Figure S2. MCD for pyrostatin B 6

Table S4. NMR spectroscopic data of madurastatin [4] 7

Figure S3. MCD for madurastatin. 8

Table S5. NMR spectroscopic data of dichomitol [5] 9

Figure S4. MCD for dichomitol 9

Table S6. NMR spectroscopic data of samoquasine A [6] 10

Figure S5. MCD for samoquasine A 10

Table S7. Spectroscopic data of palmarumycin B6 [7] 11

Figure S6. MCD for palmarumycin B6 11

Table S8. NMR spectroscopic data of nocarbenzoxazole G [8] 12

Figure S7. MCD for nocarbenzoxazole G 12

Table S9. NMR spectroscopic data of hetiamacin A [9] 13

Figure S8. MCD for hetiamacin A 14

Table S10. NMR spectroscopic data of uniflorin [10] 15

Figure S9. MCD for uniflorin 15

Table S11. NMR spectroscopic data of altechromone A [11] 16

Figure S10. MCD for altechromone A 16

Table S12. NMR spectroscopic data of aruncin B [12] 17

Figure S11. MCD for aruncin B 17

Figure S12. Sixteen possible diastereomers of dichomitol with fixed R-chirality at the C11 carbon. Natural diastereomer of dichomitol is **15a** 18

Table S13. Experimental [5] and DFT-calculated ^{1}H NMR chemical shifts for sixteen possible diastereomers of dichomitol. Natural diastereomer of dichomitol is **15a**[a]................................19

Table S14. Experimental [5] and DFT-calculated ^{13}C NMR chemical shifts for sixteen possible diastereomers of dichomitol. Natural diastereomer of dichomitol is **15a**[a]................................20

Table S15. Experimental [7] and DFT-calculated ^{13}C NMR chemical shifts of the top six CASE-generated structures for palmarumycin B6 22

Table S16. Experimental [8] and DFT-calculated ^{13}C NMR chemical shifts for the top four CASE-generated structures and originally proposed structure for norcarbenzoxazole G[a]......23

Table S17. Experimental [9] and DFT-calculated ^{13}C NMR chemical shifts of the top three CASE-generated structures and originally proposed structure for hetiamacin A[a]...................24

Table S18. Summary of proposed and revised structures 25

References..27

Table S1. NMR spectroscopic data of Macahydantoin B [1]

Label	δC	XHn	δH	COSY	H to C HMBC
C 1	188.2	C			
C 2	175.6	C			
C 3	74.8	C			
C 4	27.8	CH2	1.98	2.13	C 3
C 4	27.8	CH2	1.89		
C 5	25.4	CH2	2.13	1.98, 4.21	
C 6	48.6	CH2	3.53		
C 6	48.6	CH2	4.21	2.13	C 3
C 7	63.9	CH2	3.91		
C 7	63.9	CH2	3.7		C 3, C 2
C 8	45.2	CH2	5		C 10, C 14, C 2, C 1
C 8	45.2	CH2	4.95, 5.0		
C 9	137	C			
C 10	113.5	CH	6.95		C 12, C 14
C 11	159.6	C			
C 12	113.4	CH	6.79	7.21	
C 13	129.5	CH	7.21	6.79, 7.00	C 9, C 11
C 14	120.3	CH	7	7.21	
C 15	55.2	CH3	3.77		C 11

Table S2. NMR spectroscopic data of clionastatin B [2]

Label	δC	CHn	δH	M	COSY	H to C HMBC
C 1	67.5	CH	4.2	d	4.71	C 19, C 2, C 4, C 3
C 2	61.3	CH	4.71	u	4.20, 6.06	C 10, C 1, C 4, C 3
C 3	132.8	CH	6.06	u	4.71, 6.46	C 2, C 1, C 4, C 5
C 4	128.6	CH	6.46	d	6.06	C 10, C 2, C 6, C 3
C 5	149.3	C				
C 6	130.1	CH	6.53	s		C 10, C 4, C 8, C 5, C 7
C 7	181.6	C				
C 8	138.5	C				
C 9	154.9	C				
C 10	52.3	C				
C 11	27.3	CH2	2.08	u		
C 11	27.3	CH2	2.69	u		C 12, C 10, C 8, C 9
C 12	35	CH2	1.92	u		C 18, C 11, C 13, C 14
C 12	35	CH2	1.48	u		
C 13	45.5	C				
C 14	50.1	CH	3.99	s		C 18, C 13, C 16, C 8, C 9, C 7
C 15	205.9	C				
C 16	134	CH	6.31	d	7.4	C 13, C 14, C 15
C 17	170.5	CH	7.4	d	6.31	C 18, C 13, C 15
C 18	27	CH3	1.32	s		C 12, C 14, C 17
C 19	43.6	CH2	3.76	u		C 10, C 1, C 5, C 9
C 19	43.6	CH2	4.24	u		

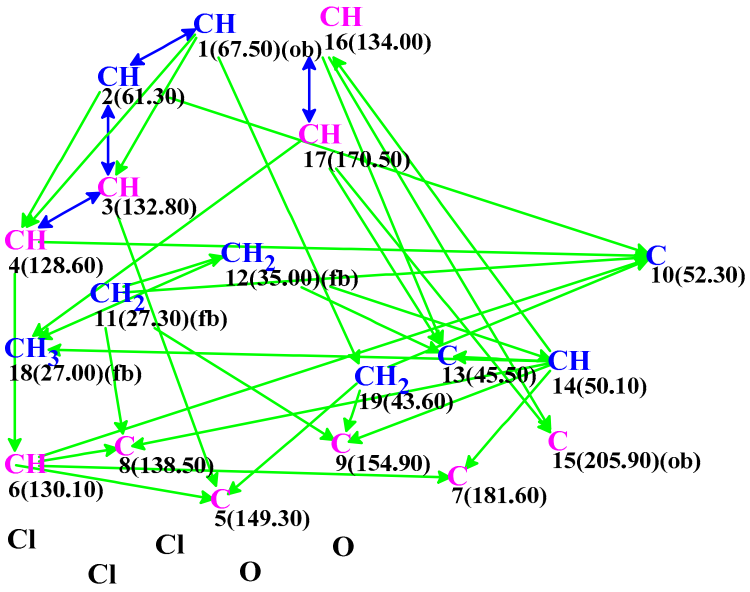

Figure S1. MCD for clionastatin B. Molecular connectivity diagram (MCD) for clionastatin B is based on COSY (blue arrows) and HMBC (green arrows) correlations. The hybridizations of carbon atoms are marked by corresponding colors: sp2—violet, sp3—blue. Labels “ob” and “fb” are set by the program to carbon atoms, for which neighboring with heteroatom is either obligatory (ob) or forbidden (fb).

Table S3. NMR spectroscopic data of pyrostatin B [3]

Label	δC	XHn	δH	H to C HMBC
C 2	161.2	C		
C 3	38	CH2	3.46	
C 3	38	CH2	3.3	C 4, C 5, C 2
C 4	22.1	CH2	2.1	C 3, C 5, C 7
C 5	53.9	CH	4.07	C 4, C 3, C 2, C 7
C 6	18.9	CH3	2.24	C 2
C 7	177.4	C		

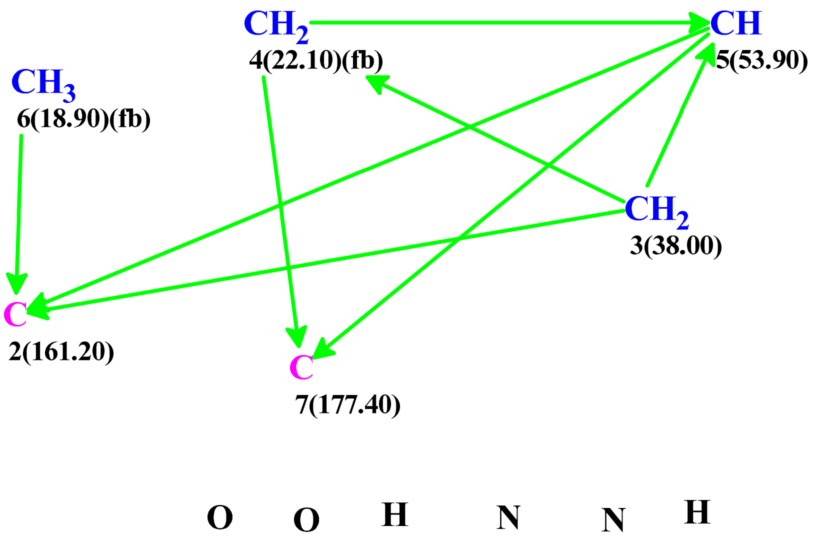

Figure S2. MCD for pyrostatin B

Table S4. NMR spectroscopic data of madurastatin [4]

C/X Label	δC	XHn	δH	M	COSY	H to C HMBC
C 1	159.1	C				
C 2	116.6	CH	7.01	u	7.47	C 6
C 3	134.1	CH	7.47	u	6.95, 7.01	C 1
C 4	119.1	CH	6.95	u	7.47, 7.65	
C 5	128.1	CH	7.65	u	6.95	C 1, C 7
C 6	109.9	C				
C 7	165.9	C				
C 9	67.4	CH	5.01	u	4.65	C 7
C 10	69.4	CH2	4.65	u	5.01	C 7, C 11
C 10	69.4	CH2	4.52	u		
C 11	170.2	C				
C 13	42.2	CH2	3.67	u		
C 13	42.2	CH2	3.75	u	8.52	
C 14	168.4	C				
C 16	34.6	CH2	3.25	u	2.52, 7.93	
C 17	32	CH2	2.52	u	3.25	C 18
C 18	170.9	C				
C 20	47	CH2	3.47	u	1.58	C 18
C 21	22.8	CH2	1.58	u	1.47, 3.47	
C 22	30.2	CH2	1.47	u	1.58, 2.87	
C 22	30.2	CH2	1.4	u		
C 23	63.7	CH	2.87	u	1.47	C 26
C 25	34.2	CH3	2.21	u		C 23
C 26	173.5	C				
C 28	49.4	CH	4.32	u	1.67, 8.11	
C 29	27.8	CH2	1.67	u	1.90, 4.32	
C 29	27.8	CH2	1.89	u		
C 30	20.4	CH2	1.9	u	1.67, 3.46	
C 31	51.2	CH2	3.46	u	1.9	165
C 33	165	C				
N 1		N				
N 2		NH	8.52	u	3.75	168.40, 170.20
N 3		NH	7.93	u	3.25	168.4
N 4		N				
N 5		NH				
N 6		NH	8.11	u	4.32	165.00, 173.50
N 7		N				

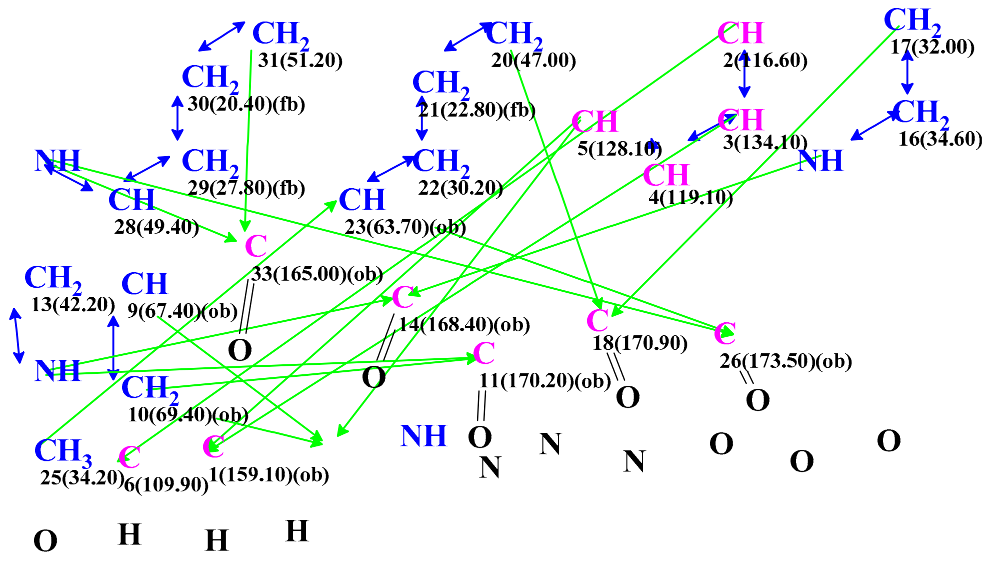

Figure S3. MCD for madurastatin.

Table S5. NMR spectroscopic data of dichomitol [5]

Label	δC	XHn	δH	C to H HMBC
C 1	36	CH2	1.44	
C 1	36	CH2	1.35	
C 2	45.5	CH	2.4	
C 3	45.9	C		
C 4	36.1	CH2	1.87	
C 4	36.1	CH2	1.85	
C 5	25.1	CH2	2.64	
C 5	25.1	CH2	2.78	
C 6	129.1	C		
C 7	145.8	C		
C 8	74.3	CH	4.13	C 10, C 2, C 3, C 9, C 6, C 7
C 9	50.5	CH	2.33	
C 10	40.8	CH2	1.73	
C 10	40.8	CH2	1.27	
C 11	45.1	C		
C 12	72.1	CH2	3.46	
C 12	72.1	CH2	3.48	C 1, C 10, C 11
C 13	22.7	CH3	0.99	C 1, C 10, C 11
C 14	20.3	CH3	1.07	C 4, C 2, C 3, C 7
C 15	59	CH2	4.22	
C 15	59	CH2	4.2	C 5, C 6, C 7

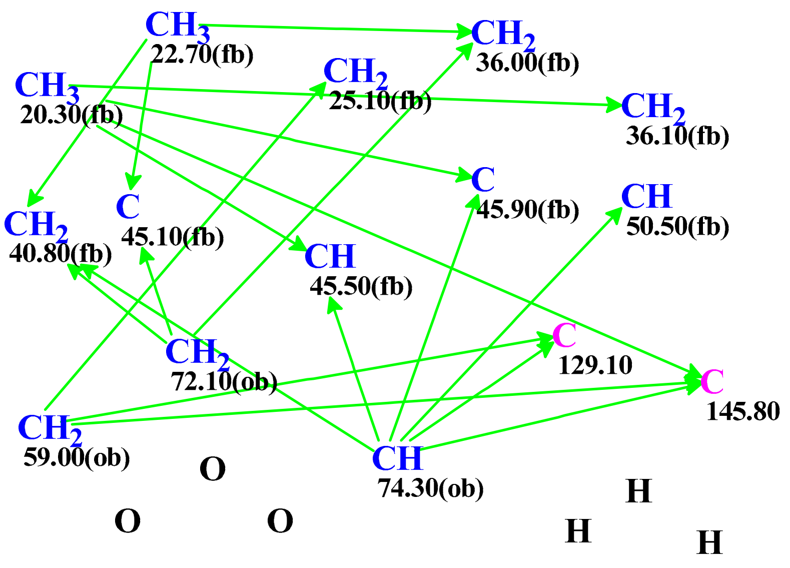

Figure S4. MCD for dichomitol

Table S6. NMR spectroscopic data of samoquasine A [6]

Label	δC	XHn	δH	COSY	H to CHMBC
C 1	150.5	CH	9.6		C 3, C 12, C 11
C 2	164	C			
C 3	118.7	C			
C 4	136	CH	7.55	7.3	C 6, C 12, C 2
C 5	101.6	CH	7.3	7.55	C 3, C 4
C 6	123.6	C			
C 7	125	CH	8.4	7.71	C 12, C 11
C 8	128.7	CH	7.71	7.87, 8.40	C 6, C 10
C 9	132.8	CH	7.87	7.71, 8.12	C 7, C 11
C 10	130.4	CH	8.12	7.87	C 6, C 8
C 11	148.5	C			
C 12	144.3	C			

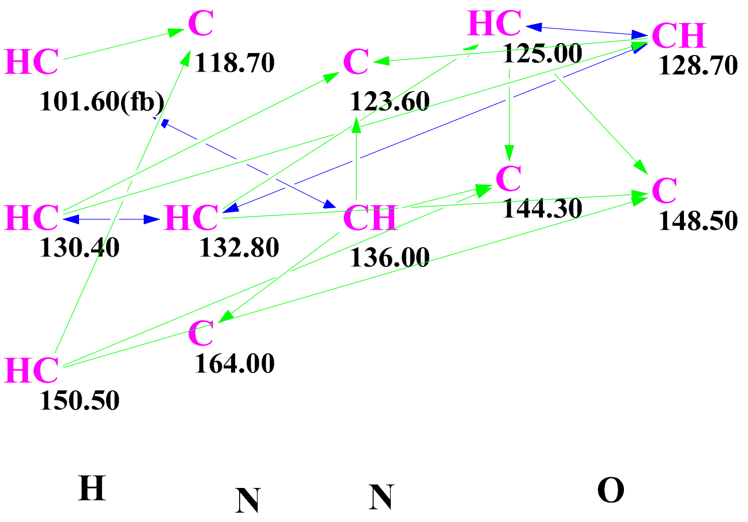

Figure S5. MCD for samoquasine A

Table S7. Spectroscopic data of palmarumycin B6 [7]

Label	δC	XHn	δH
C 1	116.3	C	
C 2	124.2	C	
C 3	158.1	C	
C 4	139.6	C	
C 5	117.2	CH	7.42
C 6	137.1	CH	7.7
C 7	34.3	CH2	2.88
C 8	29.5	CH2	2.49
C 9	203.4	C	
C 10	98.2	C	
C 12	147.3	C	
C 13	113.4	C	
C 16	134.4	C	
C 17	121.2	CH	7.54
C 18	109.6	CH	6.97
C 19	127.7	CH	7.46
		OH	11.846

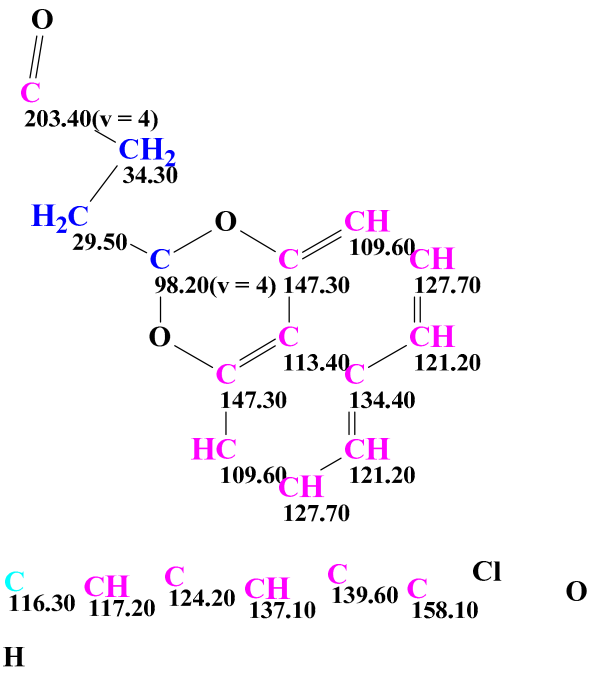

Figure S6. MCD for palmarumycin B6

Table S8. NMR spectroscopic data of nocarbenzoxazole G [8]

Label	δC	XHn	δH
C 1	165.700	C	
C 2	118.500	CH	7.690
C 3	143.200	C	
C 4	140.200	C	
C 5	65.200	CH2	4.730
C 6	125.400	CH	7.390
C 7	111.400	CH	7.610
C 8	151.300	C	
C 9	119.300	C	
C 10	149.700	C	
C 11	111.700	CH	7.770
C 12	152.200	C	
C 13	122.900	CH	7.740
C 14	117.000	CH	6.970
C 15	56.700	CH3	3.990

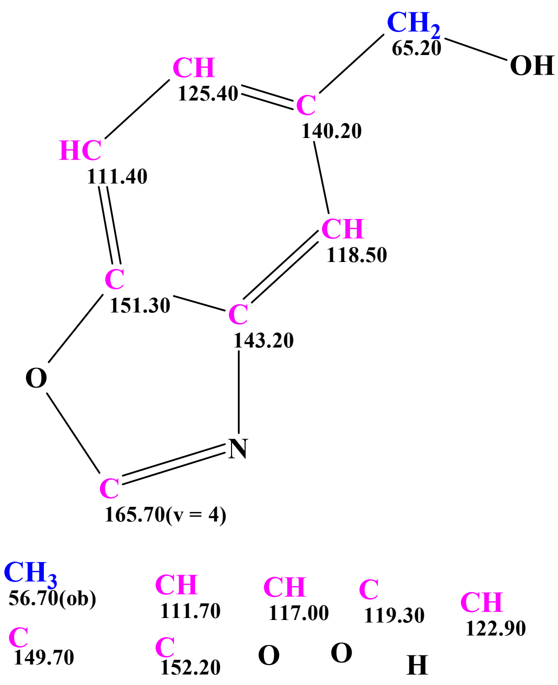

Figure S7. MCD for nocarbenzoxazole G

Table S9. NMR spectroscopic data of hetiamacin A [9]

Label	δC	XHn	δH	H to C HMBC
C 1	109.2	C		
C 2	141.5	C		
C 3	163.2	C		
C 4	118.3	CH	6.72	C 10, C 1, C 5, C 6, C 2
C 5	116.5	CH	6.78	C 1, C 4, C 6, C 3
C 6	137.3	CH	7.39	C 5, C 4, C 2, C 3
C 8	82.6	CH	4.6	C 10, C 12, C 11, C 2, C 9
C 9	170.9	C		
C 10	30.7	CH2	3.03	C 11, C 8, C 1, C 4, C 2
C 10	30.7	CH2	2.84	C 11, C 8, C 1, C 4, C 2
C 11	50.1	CH	4.3	C 13, C 10, C 12, C 8, C 19
C 12	40.6	CH2	1.39	C 14, C 17, C 13, C 11, C 8
C 12	40.6	CH2	1.76	C 14, C 17, C 13, C 11, C 8
C 13	25.7	CH	1.63	C 14, C 17, C 12, C 11
C 14	21.8	CH3	0.89	C 17, C 13, C 12
C 17	23.6	CH3	0.92	C 14, C 13, C 12
C 19	172.8	C		
C 21	81.2	CH	3.75	C 23, C 25, C 24, C 19
C 23	58.8	CH	2.91	C 28, C 25, C 24, C 21, C 29
C 24	79.2	CH2	4.5	C 23, C 21
C 24	79.2	CH2	4.13	C 23, C 21
C 25	70.3	CH	3.22	C 28, C 23, C 21, C 19
C 28	38	CH2	2.26	C 23, C 25, C 29
C 28	38	CH2	2.22	C 23, C 25, C 29
C 29	176.6	C		
		NH2	5.19	
		NH2	5.565	
		NH	11.624	
		OH	2.706	
		OH	7.035	
		NH	11.052	

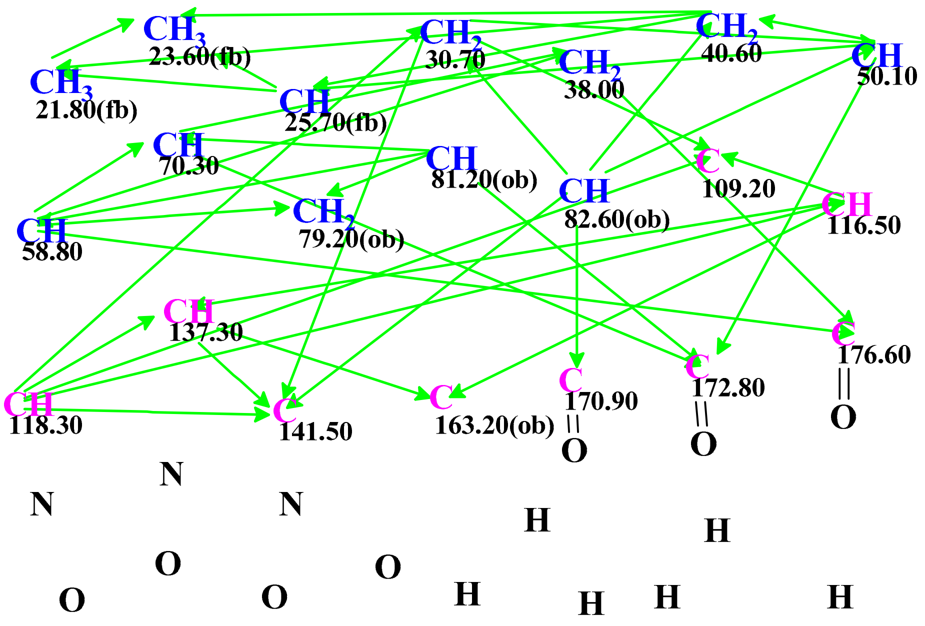

Figure S8. MCD for hetiamacin A

Table S10. NMR spectroscopic data of uniflorin [10]

Label	δC	XHn	δH
C 1	73.6	CH	3.14
C 3	81.2	CH	20
C 4	65.3	CH2	3.76
C 5	79.9	CH	3.81
C 6	72.5	CH	2.76
C 7	78.1	CH	4.18
C 8	74.2	CH	4.35
C 9	60	CH2	3.04

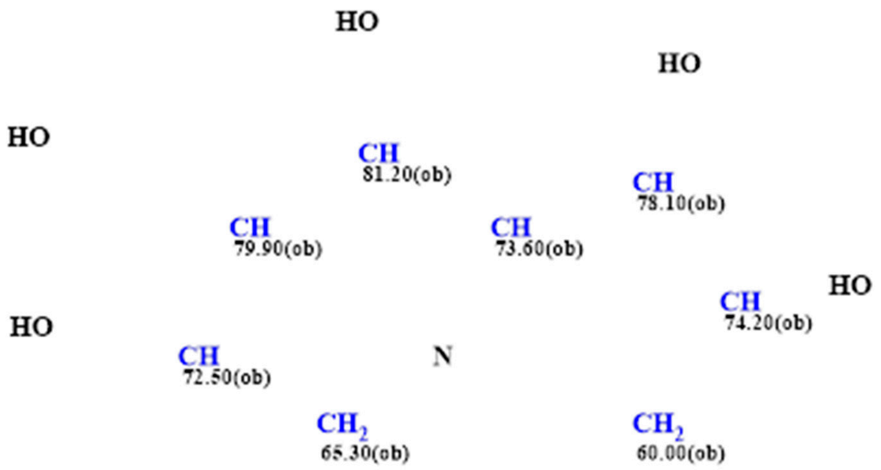

Figure S9. MCD for uniflorin

Table S11. NMR spectroscopic data of altechromone A [11]

C Label	C	CHn	H
C 1	116.5	C	
C 2	159.1	C	
C 3	141.4	C	
C 4	100.5	CH	6.65
C 5	110.7	CH	6.61
C 6	160.9	C	
C 7	114.2	CH	5.96
C 8	163.8	C	
C 9	178.3	C	
C 12	19.3	CH3	2.26
C 14	22.4	CH3	2.64
		OH	10.55

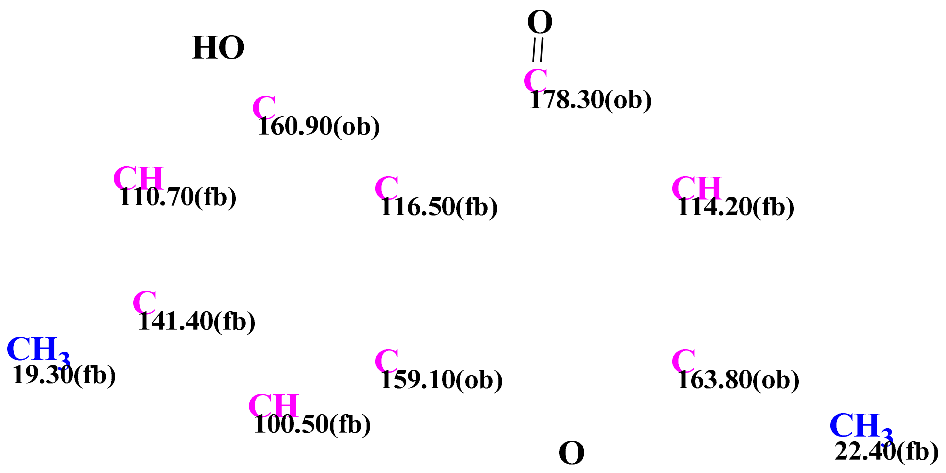

Figure S10. MCD for altechromone A

Table S12. NMR spectroscopic data of aruncin B [12]

C Label	δC	CHn	δH	M
C 2	64.2	CH2	3.64	u
C 2	64.2	CH2	3.76	u
C 3	76.8	CH	4.27	u
C 4	132.3	C		
C 5	143	CH	7.46	u
C 6	147.5	C		
C 7	124.9	CH	5.57	s
C 8	71.3	C		
C 9	30.3	CH3	1.5	s
C 10	66.8	CH2	3.57	q
C 11	15.8	CH3	1.22	t
C 12	170.8	C		

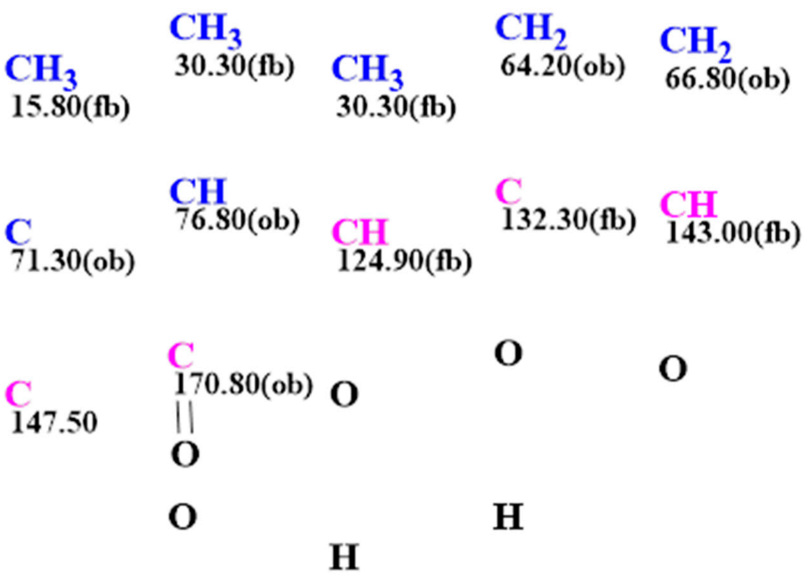

Figure S11. MCD for aruncin B

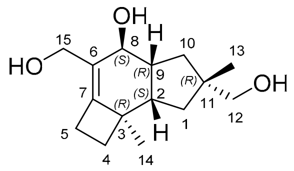

15a

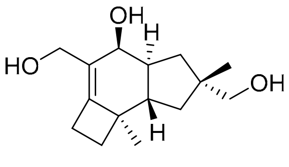

15b

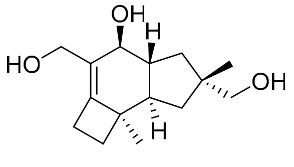

15c

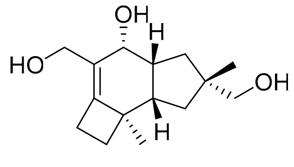

15d

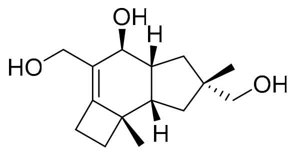

15e

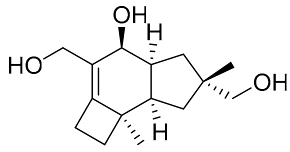

15f

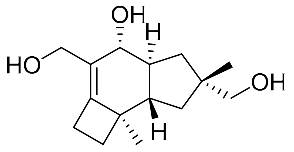

15g

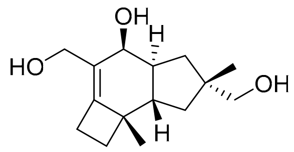

15h

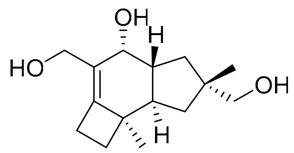

15i

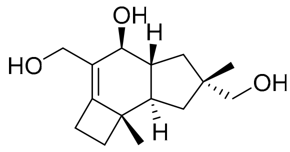

15j

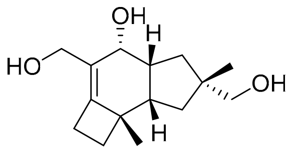

15k

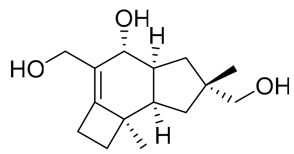

15l

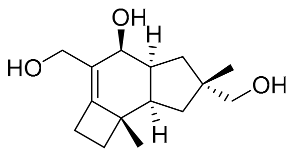

15m

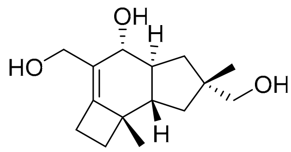

15n

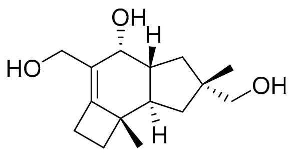

15o

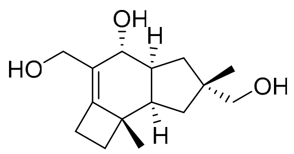

15p

Figure S12. Sixteen possible diastereomers of dichomitol with fixed R-chirality at the C11 carbon. Natural diastereomer of dichomitol is **15a**

Table S13. Experimental [5] and DFT-calculated ^{1}H NMR chemical shifts for sixteen possible diastereomers of dichomitol. Natural diastereomer of dichomitol is **15a**[a]

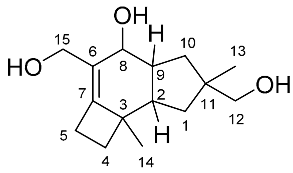

Protons	Exper.	**15a**	**15b**	**15c**	**15d**	**15e**	**15f**	**15g**	**15h**
1a	1.35	1.24	1.20	0.88	1.23	1.09	1.14	1.18	1.21
1b	1.44	1.52	1.46	1.69	1.73	1.46	1.60	1.42	1.30
2	2.40	2.32	1.87	1.46	2.22	2.04	2.10	1.80	1.93
4a	1.87	1.81	1.79	1.68	1.89	1.76	1.57	1.75	1.63
4b	1.85	1.74	1.64	1.45	1.66	1.58	2.01	1.57	1.46
5a	2.64	2.56	2.65	2.45	2.55	2.44	2.50	2.47	2.39
5b	2.78	2.74	2.91	2.80	2.85	2.84	2.72	2.87	2.90
8	4.13	4.07	4.13	4.04	4.10	4.13	4.40	4.08	4.04
9	2.33	2.25	1.81	1.60	2.30	2.05	2.65	1.77	1.52
10a	1.27	1.28	1.51	1.23	1.33	1.51	1.58	1.00	1.27
10b	1.73	1.62	1.59	1.43	1.85	1.80	1.30	1.98	1.48
12a	3.48	3.36	3.29	3.28	3.39	3.31	3.33	3.25	3.26
12b	3.46	3.35	3.27	3.26	3.32	3.29	3.29	3.21	3.23
13	0.99	0.96	0.96	0.98	0.95	0.96	0.99	0.96	0.99
14	1.07	1.02	1.08	1.19	1.29	1.32	1.12	1.17	1.30
15a	4.20	4.08	3.95	4.12	3.85	3.95	4.08	4.00	3.87
15b	4.22	4.11	4.16	4.09	4.08	4.10	4.09	4.16	4.03
RMSD, ppm		**0.086**	0.225	0.357	0.157	0.198	0.223	0.258	0.305
max_dev, ppm		**0.12**	0.53	0.94	0.35	0.36	0.43	0.60	0.81
r		**0.9991**	0.9841	0.9664	0.9922	0.9875	0.9799	0.9813	0.9773
n		292	174	168	228	161	476	181	141

Protons	Exper.	**15i**	**15j**	**15k**	**15l**	**15m**	**15n**	**15o**	**15p**
1a	1.35	0.82	1.09	0.96	1.08	1.30	1.25	1.23	1.24
1b	1.44	1.70	1.57	2.05	1.53	1.59	1.28	1.59	1.59
2	2.40	1.83	1.73	2.01	2.03	2.19	1.52	2.49	2.26
4a	1.87	1.62	1.73	1.91	1.84	1.89	1.68	1.77	1.81
4b	1.85	1.47	1.56	1.64	1.58	1.67	1.45	1.65	1.73
5a	2.64	2.40	2.47	2.55	2.46	2.57	2.44	2.63	2.55
5b	2.78	2.89	2.87	2.86	2.84	2.85	2.80	2.90	2.74
8	4.13	4.06	4.06	4.19	4.03	4.08	4.04	4.20	4.06
9	2.33	1.57	1.87	2.53	2.14	2.34	1.53	1.97	2.17
10a	1.27	1.15	1.26	1.49	1.35	1.41	0.98	1.41	1.07
10b	1.73	1.44	1.71	2.34	1.74	1.76	1.74	1.88	1.91
12a	3.48	3.27	3.24	3.52	3.26	3.37	3.28	3.38	3.33
12b	3.46	3.27	3.22	3.09	3.23	3.30	3.24	3.21	3.33
13	0.99	0.98	0.98	0.88	1.00	1.04	0.99	0.92	1.03
14	1.07	1.29	1.18	1.17	1.32	1.29	1.21	1.09	1.02
15a	4.20	3.87	4.00	4.02	3.92	3.85	4.07	3.94	4.08
15b	4.22	4.04	4.12	4.27	4.15	4.08	4.11	4.25	4.10
RMSD, ppm		0.334	0.252	0.286	0.192	0.147	0.335	0.159	0.122
max_dev, ppm		0.76	0.67	0.61	0.37	0.35	0.88	0.36	0.20
r		0.9711	0.9826	0.9649	0.9897	0.9945	0.9720	0.9900	0.9961
n		141	162	127	467	267	162	162	265

Table S14. Experimental [5] and DFT-calculated ^{13}C NMR chemical shifts for sixteen possible diastereomers of dichomitol. Natural diastereomer of dichomitol is **15a**[a]

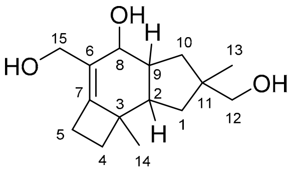

Carbons	Exper.	**15a**	**15b**	**15c**	**15d**	**15e**	**15f**	**15g**	**15h**
1	36.0	35.3	35.0	34.8	36.5	37.0	39.8	35.6	34.6
2	45.5	47.4	44.6	49.6	46.1	49.7	45.6	51.7	42.0
3	45.9	48.3	50.3	52.1	48.3	47.7	47.8	49.8	51.6
4	36.1	35.3	32.1	26.4	35.4	28.4	28.4	32.5	25.3
5	25.1	25.3	30.4	30.3	27.1	27.8	25.6	29.0	30.6
6	145.8	147.1	150.9	145.9	154.5	146.8	145.3	150.5	151.7
7	129.1	132.3	127.9	132.1	131.7	126.9	129.7	128.6	131.8
8	74.3	75.1	69.9	75.6	69.6	76.1	69.6	79.1	68.8
9	50.5	51.5	45.5	52.2	46.6	47.9	46.3	47.5	49.5
10	40.8	39.9	34.0	36.3	35.9	41.2	37.1	40.7	34.9
11	45.1	47.8	43.7	45.5	47.2	45.7	45.8	45.5	45.7
12	72.1	70.7	71.0	70.7	70.8	70.5	68.5	71.0	70.9
13	22.7	20.0	24.3	24.1	21.5	24.8	21.5	24.5	24.3
14	20.3	18.6	16.5	24.1	22.4	24.9	22.8	17.4	25.4
15	59.0	59.0	62.3	60.2	61.6	63.0	59.9	62.9	63.1
RMSD, ppm		**1.71**	3.77	3.92	3.39	3.15	3.20	3.30	4.84
max_dev, ppm		**3.2**	6.8	9.7	8.7	7.7	7.7	6.2	10.8
r		**0.9992**	0.9950	0.9942	0.9967	0.9962	0.9963	0.9966	0.9916
n		292	174	168	228	161	476	181	141

Carbons	Exper.	**15i**	**15j**	**15k**	**15l**	**15m**	**15n**	**15o**	**15p**
1	36.0	34.7	35.6	34.8	37.9	36.4	34.7	37.5	35.6
2	45.5	43.1	52.9	45.5	48.9	46.8	48.4	46.9	47.9
3	45.9	51.7	49.6	47.8	47.8	47.9	52.1	51.6	48.2
4	36.1	25.3	32.4	28.5	28.4	36.1	26.4	32.0	35.3
5	25.1	30.7	29.0	28.3	27.4	26.6	30.3	30.8	25.3
6	145.8	152.0	150.7	145.1	148.8	155.0	145.8	151.8	147.3
7	129.1	131.6	128.5	126.4	127.3	132.1	132.1	127.8	132.1
8	74.3	69.0	79.3	67.5	75.3	68.0	75.8	71.2	75.5
9	50.5	47.9	46.6	43.8	47.4	46.8	53.6	44.6	52.1
10	40.8	34.9	40.3	36.7	41.0	35.6	36.7	34.0	41.0
11	45.1	45.7	45.3	45.7	45.4	47.9	45.5	45.6	48.0
12	72.1	70.7	70.6	70.4	69.8	68.1	70.7	73.8	67.8
13	22.7	24.3	24.4	25.8	24.1	22.2	24.4	24.1	22.4
14	20.3	25.4	17.3	23.9	25.5	22.5	24.2	15.5	18.5
15	59.0	63.0	62.9	62.1	63.0	61.4	60.2	62.6	59.0
RMSD, ppm		4.84	3.57	3.87	3.24	3.80	3.92	4.13	1.94
max_dev, ppm		10.8	7.4	7.6	7.7	9.2	9.7	6.8	4.3
r		0.9916	0.9958	0.9945	0.9960	0.9956	0.9942	0.9939	0.9988
n		141	162	127	467	267	162	162	265

[a] Prior to chemical shift analysis the conformational analysis of all molecules was carried out using Spartan'20 program [13] and MMFF force-field. Conformational ensembles were then energy optimized using DFT calculations at the B3LYP/6-31+G(d,p) level of theory and chemical shifts were computed at

the mPW1PW91/6-311+G(2d,p) level with the inclusion of SMD solvent model for chloroform. DFT calculations were carried out using Gaussian16 program [14]. ^{1}H and ^{13}C NMR chemical shifts were determined from Boltzmann averaged isotropic values using the following scaling factors: ^{1}H slope: -1.0936 and intercept: 31.8018; ^{13}C slope: -1.0533 and intercept: 186.5242 [15]; RMSD – root-mean-square deviation; max_dev – maximum deviation, *r* – correlation coefficient, n – number of conformations;

Table S15. Experimental [7] and DFT-calculated ^{13}C NMR chemical shifts of the top six CASE-generated structures for palmarumycin B6[a]

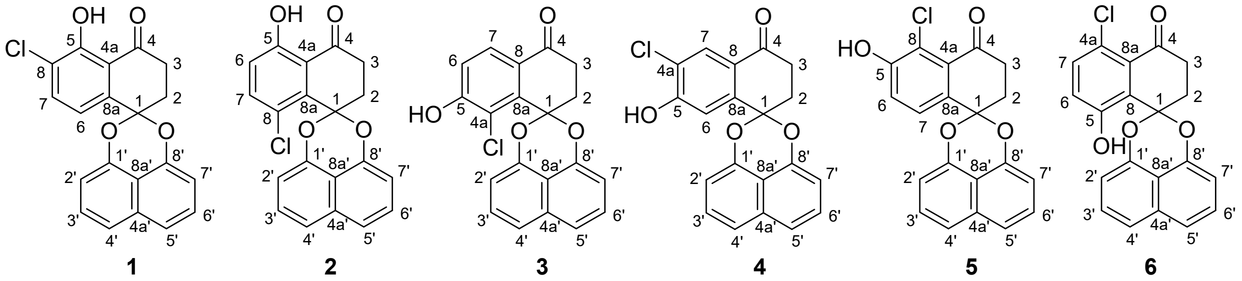

Carbons	Exper.	**1**	**2**	**3**	**4**	**5**	**6**
C 1	98.2	101	101.5	102.1	101.1	101.4	105.8
C 2	29.5	29.8	30.5	31	30.2	29.3	29
C 3	34.3	34.4	34.1	33.7	34	34.7	32.6
C 4	203.4	204.7	204.8	194.7	194.3	194.4	193.6
C 4a	116.3	116.6	117.2	117	118.6	117.4	124.6
C 5	158.1	160.3	162.4	156.8	155.8	153.7	154.8
C 6	117.2	116.3	120.1	116.8	114.7	119.7	122.7
C 7	137.1	137.8	140.7	130.4	131.3	127.8	135.3
C 8	124.2	122.8	122.9	126.2	125.3	129.2	124.1
C 8a	139.6	140.9	139	139.5	143.2	134.7	132.1
C 1'	147.3	148.4	148.1	148.1	148.5	148.8	147.3
C 2'	109.6	109.4	109.2	109.3	109.4	109.4	110.4
C 3'	127.7	128.5	128.4	128.4	128.4	128.5	128.6
C 4'	121.2	120.5	120.2	120.2	120.4	120.4	121.5
C 4a'	134.4	134.2	134.2	134.2	134.2	134.2	134.1
C 5'	121.2	120.5	120.2	120.2	120.4	120.4	121.5
C 6'	127.7	128.5	128.4	128.4	128.4	128.5	128.6
C 7'	109.6	109.4	109.2	109.3	109.4	109.4	110.4
C 8'	147.3	148.4	148.1	148.1	148.5	148.8	147.3
C 8a'	113.4	114.1	113.3	113.5	114.2	114.3	114.3
RMSD,[b] ppm		**1.11**	1.75	2.73	2.85	3.62	4.06
max_dev,[c] ppm		**2.8**	4.3	8.7	9.1	9.3	9.8
r[d]		**0.9997**	0.9991	0.9979	0.9974	0.9959	0.9944

[a] DFT computations were carried out at the ωB97-D/6-31G(d)//ωB97-D/6-31G(d) level of theory by Spartan'20 program [13]; [b] RMSD – root-mean-square deviation; [c] max_dev – maximum deviation, [d] r – correlation coefficient.

Table S16. Experimental [8] and DFT-calculated ^{13}C NMR chemical shifts for the top four CASE-generated structures and originally proposed structure for norcarbenzoxazole G[a]

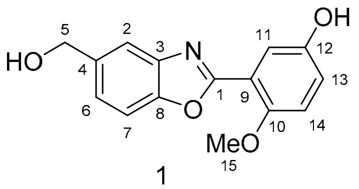

1

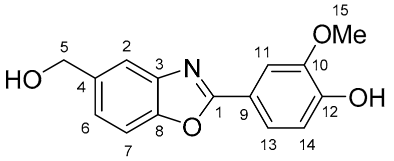

2

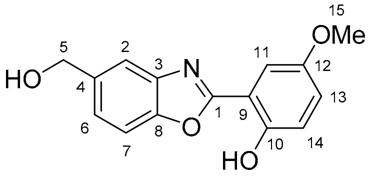

3

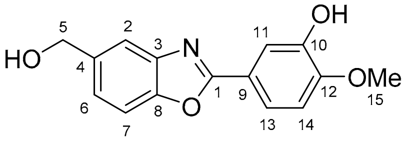

4

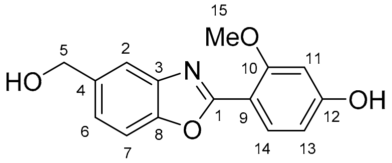

Original

Carbons	Exper.	**1**	**2**	**3**	**4**	Original
C 1	165.7	164.3	164.3	164.3	164.3	164.9
C 2	118.5	121.8	121.6	121.7	121.7	121.2
C 3	143.2	142.0	143.3	142.0	143.3	142.4
C 4	140.2	137.0	136.8	137.0	136.9	136.7
C 5	65.2	66.6	66.6	66.6	54.9	66.7
C 6	125.4	127.7	127.7	127.8	127.7	126.8
C 7	111.4	110.8	110.5	110.9	110.4	110.5
C 8	151.3	151.4	151.0	151.4	150.8	151.1
C 9	119.3	115.5	119.2	112.8	120.0	108.2
C 10	149.7	153.3	146.9	150.8	146.8	161.3
C 11	111.7	116.2	109.5	110.9	113.1	98.1
C 12	152.2	150.0	150.4	153.4	150.3	161.8
C 13	122.9	120.7	122.0	123.6	120.9	106.7
C 14	117.0	113.3	114.1	118.6	110.3	134.4
C 15	56.7	54.3	54.7	54.2	66.7	54.4
RMSD,[b] ppm		2.71	**2.01**	2.39	4.46	8.69
max_dev,[c] ppm		4.5	**3.4**	6.5	10.3	17.4
r[d]		0.9958	**0.9981**	0.9968	0.9888	0.9600

[a] DFT calculations were carried out at the mPW1PW91/6-311+G(2d,p)//B3LYP/6-31+G(d,p) level of theory with the inclusion of PCM solvent model for methanol. DFT calculations were carried out using Gaussian16 program [14]. ^{13}C NMR chemical shifts were determined from the isotropic values using the following scaling factors: slope = -1.0399 and intercept = 186.5993 [15]; [b] RMSD – root-mean-square deviation; [c] max_dev – maximum deviation, [d] *r* – correlation coefficient.

Table S17. Experimental [9] and DFT-calculated ^{13}C NMR chemical shifts of the top three CASE-generated structures and originally proposed structure for hetiamacin A[a]

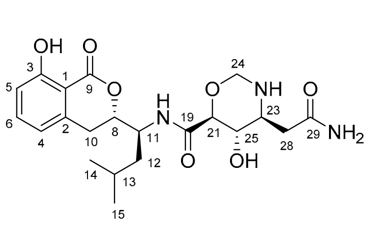
1

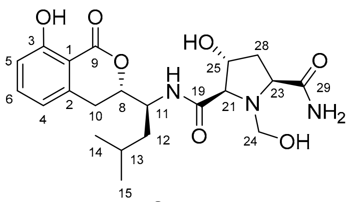
2

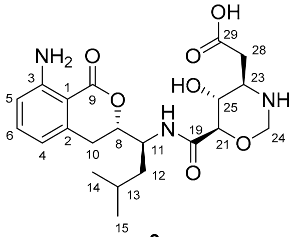
3

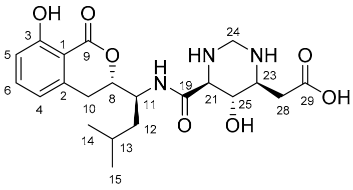
Original

Carbons	Exp	**1**	**2**	**3**	Original
C 1	109.2	108.1	107.6	104.6	107.8
C2	141.5	143	142.1	142.9	142.3
C3	163.2	163.4	163.4	152.3	163.3
C4	118.3	118	118.3	114	117.9
C5	116.5	115.2	115.8	114.2	115
C6	137.3	138.2	139	136.7	138.5
C8	82.6	81.8	82.5	81.3	80.1
C9	170.9	171.8	172	169.9	171.1
C10	30.7	30.8	31.2	32.7	30.2
C11	50.1	53.4	55.2	55.2	55.7
C12	40.6	40.9	40.1	41	41.2
C13	25.7	27.9	27.2	27.3	28
C14	21.8	21.7	20.5	22.4	20.2
C15	23.6	21.2	21.8	21	22.2
C19	172.8	174.1	177.6	175	174.2
C21	81.2	78.6	71.6	76	60.8
C23	58.8	57.5	65.8	54.4	57.4
C24	79.2	79.1	76.1	71.9	57
C25	70.3	68.5	77.8	65.6	69
C28	38	35.2	41.5	35.5	33.3
C29	176.6	175.1	180	176.5	176.5
n[b]		83	127	125	78
RMSD,[c] ppm		**1.59**	3.75	4.04	6.87
max_dev,[d] ppm		**3.3**	9.6	10.9	22.2
r[f]		**0.9996**	0.9976	0.9977	0.9927

[a] Prior to chemical shift analysis the conformational analysis of all molecules was carried out using MacroModel program [16] and OPLS3e force-field. Conformational ensembles were then energy optimized using DFT calculations at the B3LYP/6-31+G(d,p) level of theory and chemical shifts were computed at the mPW1PW91/6-311+G(2d,p) level with the inclusion of PCM solvent model for methanol. DFT calculations were carried out using Gaussian16 program [14]. ^{13}C NMR chemical shifts were determined from Boltzmann averaged isotropic values using the following scaling factors: slope = -1.0399 and intercept = 186.5993 [15]; [b] n – number of conformations; [c] RMSD – root-mean-square deviation; [d] max_dev – maximum deviation, [f] *r* – correlation coefficient.

Table S18. Summary of proposed and revised structures

PROPOSED STRUCTURE	REVISED STRUCTURE
Macahydantoin B	
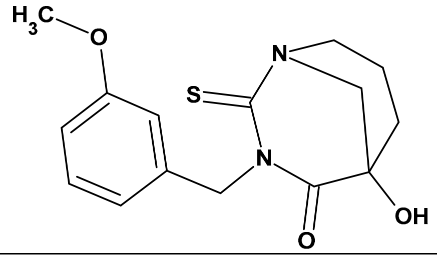	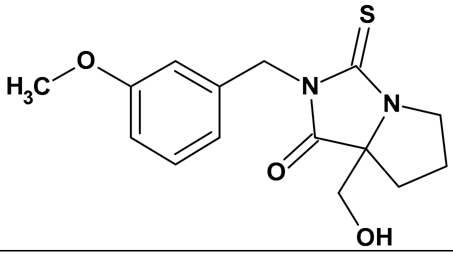
Clionastatin	
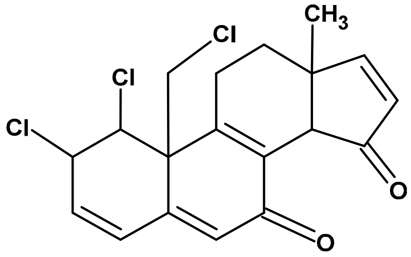	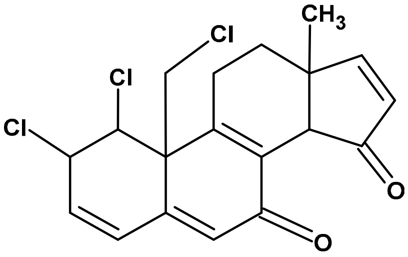 Unambiguously confirmed by CASE
Pyrostatin B	
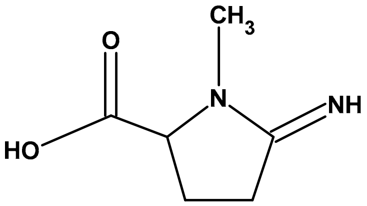	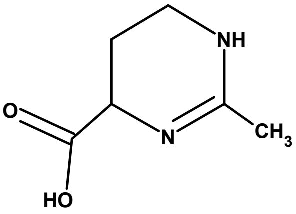
Madurastatin C	
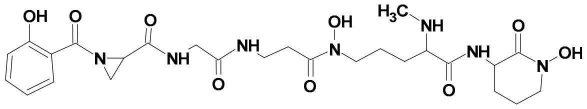	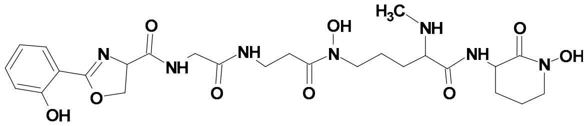
Dichomitol	
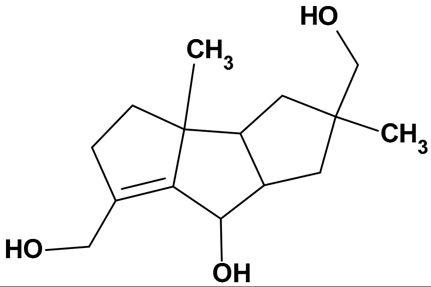	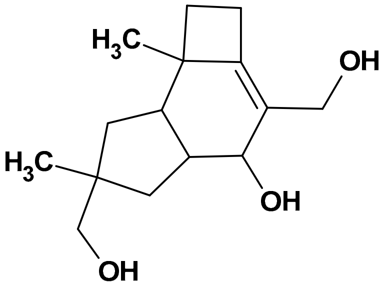
Samoquasine A	
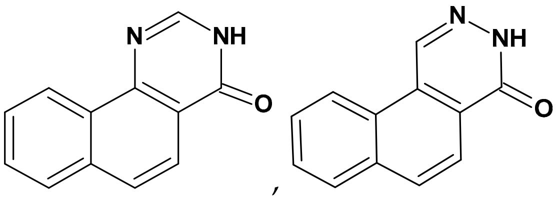	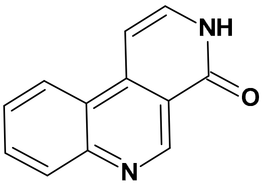

PROPOSED STRUCTURE	REVISED STRUCTURE
Palmarumicin B6	
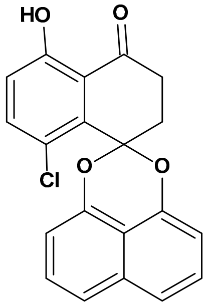	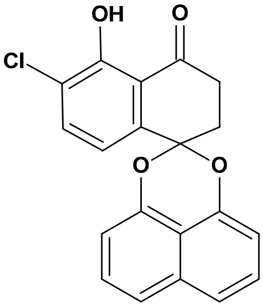
Nocarbenzoxazole G	
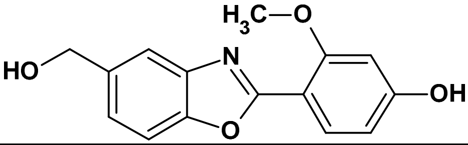	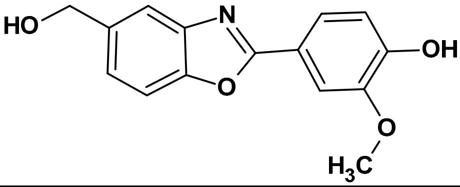
Hetiamacin A	
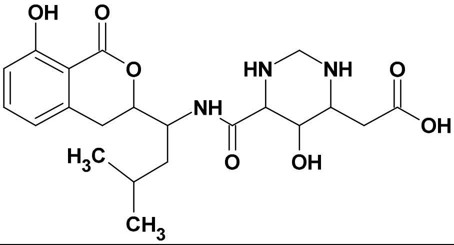	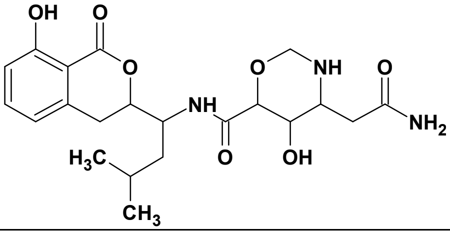
Uniflorine A	
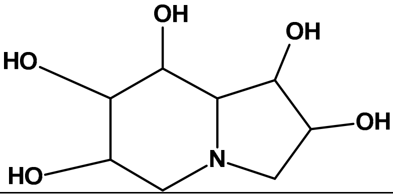	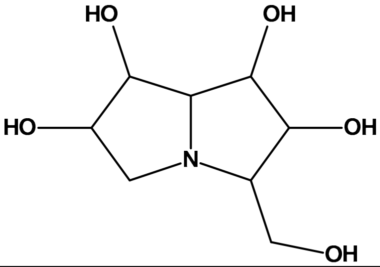
Altechromone A	
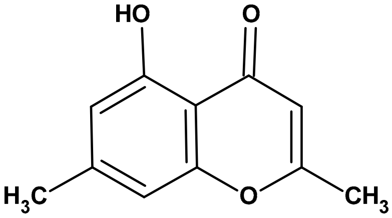	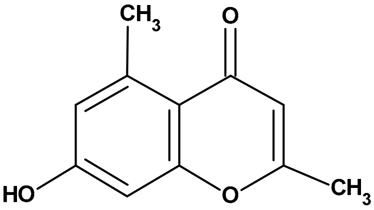
Arunicin B	
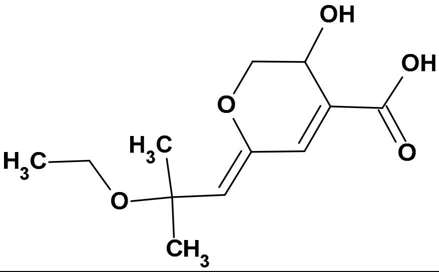	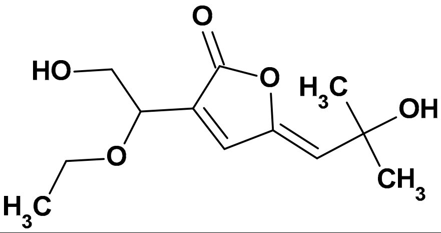

References

1. Yu, M.-Y.; Qin, X.-J.; Shao, L.-D.; Peng, X.-R.; Li, L.; Yang, H.; Qiu, M.-H., Corrigendum to "Macahydantoins A and B, two new thiohydantoin derivatives from Maca (Lepidium meyenii): Structural elucidation and concise synthesis of macahydantoin A" [Tetrahedron Lett., 58 (17) (2017) 1684–1686]. *Tetraheron Lett.* **2018,** 59, (4), 418.
2. Fattorusso, E.; Taglialatela-Scafati, O.; Petrucci, F.; Bavestrello, G.; Calcinai, B.; Cerrano, C.; Di Meglio, P.; Ianaro, A., Polychlorinated Androstanes from the Burrowing Sponge Cliona nigricans. *Org. Lett.* **2004,** 6, (10), 1633-1635.
3. Aoyama, T.; Kojima, F.; Imada, C.; Muraoka, Y.; Naganawa, H.; Okami, Y.; Takexjchi, T.; Aoyagi, T., Pyrostatins A and B, New Inhibitors of N-Acetyl-β-D-Glucosaminidase, Produced by Streptomyces sp. SA-3501. *Journal of Enzyme Inhibition* **1995,** 8, (4), 223-232.
4. Kawahara, T.; Itoh, M.; Izumikawa, M.; Sakata, N.; Tsuchida, T.; Shin-ya, K., Novel aziridine-containing peptides MBJ-0034 and MBJ-0035 from Streptosporangium sp. 32552. *J. Antibiot.* **2014,** 67, (8), 577-580.
5. Huang, Z.; Dan, Y.; Huang, Y.; Lin, L.; Li, T.; Ye, W.; Wei, X., Sesquiterpenes from the Mycelial Cultures of Dichomitus squalens. *J. Nat. Prod.* **2004,** 67, (12), 2121-2123.
6. Morita, H.; Sato, Y.; Chan, K.-L.; Choo, C.-Y.; Itokawa, H.; Takeya, K.; Kobayashi, J. i., Samoquasine A, a Benzoquinazoline Alkaloid from the Seeds of Annona squamosa. *J. Nat. Prod.* **2000,** 63, (12), 1707-1708.
7. Shan, T.; Tian, J.; Wang, X.; Mou, Y.; Mao, Z.; Lai, D.; Dai, J.; Peng, Y.; Zhou, L.; Wang, M., Bioactive Spirobisnaphthalenes from the Endophytic Fungus Berkleasmium sp. *J. Nat. Prod.* **2014,** 77, (10), 2151-2160.
8. Sun, M.; Zhang, X.; Hao, H.; Li, W.; Lu, C., Nocarbenzoxazoles A–G, Benzoxazoles Produced by Halophilic Nocardiopsis lucentensis DSM 44048. *J. Nat. Prod.* **2015,** 78, (8), 2123-2127.
9. Wu, G.; Liu, S.; Wang, T.; Jiang, Z.; Lv, K.; Wang, Y.; Sun, C., Total Synthesis of Originally Proposed and Revised Structure of Hetiamacin A. *Org. Lett.* **2018,** 20, (12), 3566-3569.
10. Matsumura, T.; Kasai, M.; Hayashi, T.; Arisawa, M.; Momose, Y.; Arai, I.; Amagaya, S.; Komatsu, Y., a-glucosidase Inhibitors From Paraguayan Natural Medicine, Ñangapiry, The Leaves Of Eugenia Uniflora. *Pharm. Biol.* **2000,** 38, (4), 302-307.
11. Königs, P.; Rinker, B.; Maus, L.; Nieger, M.; Rheinheimer, J.; Waldvogel, S. R., Structural Revision and Synthesis of Altechromone A. *J. Nat. Prod.* **2010,** 73, (12), 2064-2066.
12. Jeong, S. Y.; Jun do, Y.; Kim, Y. H.; Min, B. S.; Min, B. K.; Woo, M. H., Monoterpenoids from the aerial parts of Aruncus dioicus var. kamtschaticus and their antioxidant and cytotoxic activities. *Bioorg. Med. Chem. Lett.* **2011,** 21, (11), 3252-3256. 1. Yu, M.-Y.; Qin, X.-J.; Shao, L.-D.; Peng, X.-R.; Li, L.; Yang, H.; Qiu, M.-H., Corrigendum to "Macahydantoins A and B, two new thiohydantoin derivatives from Maca (Lepidium meyenii): Structural elucidation and concise synthesis of macahydantoin A" [Tetrahedron Lett., 58 (17) (2017) 1684–1686]. Tetraheron Lett. 2018, 59, (4), 418.
13. *Spartan '20*, Wavefunction Inc., 18401 Von Karman Avenue, Suite 370 Irvine, CA 92612 U.S.A.
14. Frisch, M. J.; Trucks, G. W.; Schlegel, H. B.; Scuseria, G. E.; Robb, M. A.; Cheeseman, J. R.; Scalmani, G.; Barone, V.; Petersson, G. A.; Nakatsuji, H.; Li, X.; Caricato, M.; Marenich, A. V.; Bloino, J.; Janesko, B. G.; Gomperts, R.; Mennucci, B.; Hratchian, H. P.; Ortiz, J. V.; Izmaylov, A. F.; Sonnenberg, J. L.; Williams; Ding, F.; Lipparini, F.; Egidi, F.; Goings, J.; Peng, B.; Petrone, A.; Henderson, T.; Ranasinghe, D.; Zakrzewski, V. G.; Gao,

J.; Rega, N.; Zheng, G.; Liang, W.; Hada, M.; Ehara, M.; Toyota, K.; Fukuda, R.; Hasegawa, J.; Ishida, M.; Nakajima, T.; Honda, Y.; Kitao, O.; Nakai, H.; Vreven, T.; Throssell, K.; Montgomery Jr., J. A.; Peralta, J. E.; Ogliaro, F.; Bearpark, M. J.; Heyd, J. J.; Brothers, E. N.; Kudin, K. N.; Staroverov, V. N.; Keith, T. A.; Kobayashi, R.; Normand, J.; Raghavachari, K.; Rendell, A. P.; Burant, J. C.; Iyengar, S. S.; Tomasi, J.; Cossi, M.; Millam, J. M.; Klene, M.; Adamo, C.; Cammi, R.; Ochterski, J. W.; Martin, R. L.; Morokuma, K.; Farkas, O.; Foresman, J. B.; Fox, D. J. *Gaussian 16* Rev. A.03, Wallingford, CT, 2016.

15. CHESHIRE. CHEmical SHift REpository with Coupling Constants Added Too. Available online: http://cheshirenmr.info (accessed on 30 March 2023).
16. *Schrödinger* Release 2021-1: MacroModel; Schrödinger, LLC: New York, NY, 2021. https://www.schrodinger.com/products/macromodel (accessed March 30, 2023).
